# Type 1 diabetes technology gap between high‐income and developing countries: Continuous glucose monitoring access remains a challenge in Brazil

**DOI:** 10.1111/dme.70116

**Published:** 2025-08-06

**Authors:** Ana Victoria Santos Castro, Karina O. Caneca, Paula M. Garcia, Veridiana Tischer, Luciana C. Theodoro, Isabella S Leão, Letícia B. Cunha, Julia B. Vieira, Ludmila N.R. Campos, Jorge L. Luescher, Joana R. Dantas, Lenita Zajdenverg, Melanie Rodacki

**Affiliations:** ^1^ Internal Medicine Department Universidade Federal do Rio de Janeiro Rio de Janeiro Brazil; ^2^ Universidade Estadual do Rio de Janeiro Rio de Janeiro Brazil; ^3^ Martagão Gesteira Institute of Pediatrics and Childcare Universidade Federal do Rio de Janeiro Rio de Janeiro Brazil

To the Editor,

We read with great interest the article by Dlugatch et al., recently published in *Diabetic Medicine*, which addresses inequalities in the access and use of diabetes technology among children and young people with type 1 diabetes (T1D) in the UK.^1^ Their findings resonate strongly with the reality faced in low‐ and middle‐income countries, particularly Brazil, where barriers to diabetes technology are even more pronounced.

Insulin pumps and continuous glucose monitoring (CGM) systems are expensive and are not currently provided by Brazil's public healthcare system (SUS), nor do private insurance routinely cover them. This creates socioeconomic inequities in diabetes care. While wealthier individuals with T1D can access and benefit from advanced technologies, those reliant on public healthcare are deprived of these tools, resulting in disparities in glycaemic control, complication risk and overall quality of care.

Even intermittent CGM (isCGM), the least costly CGM option, remains out of reach for most patients. In addition, long‐acting insulin analogues are not universally provided, and many patients are still treated with neutral protamine hagedorn (NPH) insulin in combination with rapid‐acting analogues. The absence of CGM impairs the ability to make informed insulin adjustments and to obtain key glycaemic control metrics, such as time in range (TIR), time above range (TAR) and time below range (TBR), which are increasingly recognized as important predictors of outcomes in T1D.

To evaluate the glycaemic patterns in patients without routine CGM access, we conducted an observational study involving 92 individuals (45 children and 47 adults) with T1D at a public diabetes clinic in Brazil. None of the participants had regular access to CGM; they relied exclusively on four to five daily capillary glucose measurements in a public healthcare clinic in Brazil. Their mean age, diabetes duration and HbA1c were 18.95 ± 10.06 years, 11.15 ± 8.48 years and 61 mmol/mol (7.7% ± 1.2%), respectively. Of these, 81.5% used long‐acting insulin analogues, while 18.5% were using NPH insulin with rapid‐acting analogues. After 14 days of isCGM, the mean TIR was 50.9% ± 15.1%, TAR 31.7% ± 19.1% and TBR 16.2% ± 11.4%. All metrics were outside the recommended targets. Notably, among individuals with HbA1c < 53 mmol/mol (7%), mean TBR was 21.13% ± 14.1%, with TIR and TAR at 51.9% ± 16.1% and 26.9% ± 18.1%, respectively.^2^

In a separate case, a 24‐year‐old woman with T1D using NPH and rapid‐acting insulin analogues, an HbA1c of 57 mmol/mol (7.4%) and satisfactory capillary glucose records, underwent a 15‐day real‐time CGM analysis with no recommended interventions based on CGM data. The report showed a TIR of 55%, TAR of 30% and TBR of 15%, including 10% of readings <3.9 mmol/L (<70 mg/dL) and 5% < 3.0 mmol/L (<54 mg/dL), with significant episodes of asymptomatic nocturnal hypoglycaemia—none of which had been suspected during routine care.

These data highlight the critical role of CGM in identifying suboptimal and potentially dangerous glycaemic patterns, even in patients with target‐range HbA1c levels. They further underscore how the exclusive reliance on capillary blood glucose testing can obscure the risks of hypoglycaemia. It has been previously shown that the early initiation of CGM, soon after T1D diagnosis, leads to significant improvements in outcomes and provides sustained benefits over time.^3^

Despite growing international consensus on the benefits of CGM, Brazil's National Committee for Health Technology Incorporation (CONITEC) recently advised against incorporating CGM into the public healthcare system.^4^ This decision reflects a broader challenge: ensuring equitable access to life‐saving diabetes technologies in resource‐constrained settings. As Dlugatch et al. emphasize in the UK context, the mere existence of technology is insufficient: equity in access and support is imperative.

Addressing disparities in technology use among patients with T1D is a major concern worldwide.^5^ In Brazil and other developing nations, overcoming these disparities requires coordinated policy action, public investment and inclusion of underserved populations in decision making processes. Without such measures, the promise of technology to transform diabetes care will remain inequitably distributed.^6^

## FUNDING INFORMATION

This work received a grant from CNPq (Conselho Nacional de Desenvolvimento Científico e Tecnológico).

## CONFLICT OF INTEREST STATEMENT

The authors declare no conflicts of interest.

## Data Availability

Data sharing not applicable to this article as no datasets were generated or analyzed during the current study.
